# Efficacy and safety of Chinese herbal medicine for atopic dermatitis: Evidence from eight high-quality randomized placebo-controlled trials

**DOI:** 10.3389/fphar.2022.927304

**Published:** 2022-09-27

**Authors:** Xiaoce Cai, Xiaoying Sun, Liu Liu, Yaqiong Zhou, Seokgyeong Hong, Jiao Wang, Jiale Chen, Miao Zhang, Chunxiao Wang, Naixuan Lin, Su Li, Rong Xu, Xin Li

**Affiliations:** ^1^ Department of Dermatology, Yueyang Hospital of Integrated Traditional Chinese and Western Medicine, Shanghai University of Traditional Chinese Medicine, Shanghai, China; ^2^ Institute of Dermatology, Shanghai Academy of Traditional Chinese Medicine, Shanghai, China

**Keywords:** Chinese herbal medicine, atopic dermatitis, randomized controlled trials, safety, efficacy

## Abstract

**Background:** The use of Chinese herbal medicine (CHM) for the treatment of atopic dermatitis (AD) has gained attention. This quantitative study systematically evaluated the efficacy and safety of CHM for the treatment of AD in eight high-level clinical trials, resulting in a high level of clinical evidence.

**Methods:** Several databases were searched, including PubMed, Embase, Cochrane Library, Web of Science, China National Knowledge Infrastructure (CNKI), the Chongqing VIP Chinese Science (VIP), and Wanfang Database. High-quality randomized controlled trials (RCTs) comparing CHM with placebo were included. The 95% confidence interval (CI) of the risk ratio (RR) was calculated using software (RevMan 5.3) and a meta-analysis was performed. Evidence level evaluation using GRADE Profiler 3.6.

**Results:** In total, 662 patients (322 in the experimental group and 340 in the control group) were included. The response rate of the Eczema Area and Severity Index (EASI) −90 was higher in the CHM group than in the placebo group (RR, 3.72; 95% CI, 1.76 to7.83; *p* = 0.01). Furthermore, the scoring of atopic dermatitis (SCORAD) (RR, −10.20), body surface area (BSA) (RR, −2.01), surface damage score (RR, −2.25), visual analog scale (VAS) (RR, −1.90), and sleep score (RR, −2.16), improvement of investigator’s global assessment (IGA) (RR, 2.94) improved in the CHM group. The results showed no statistical difference between CHM and placebo (MD, −0.47; 95% CI, −1.30, 0.37; *p* = 0.27) in improving the Dermatology Life Quality Index (DLQI) or children’s DLQI (CDLQI). There was also no significant difference in the IgE level between the two groups (MD, −62.76; 95% CI, −809.58, 684.05; *p* = 0.87). However, the adverse events (AEs) rate was slightly higher in patients treated with CHM than in those treated with placebo (RR, 1.42; 95% CI, 1.06–1.90; *p* = 0.02).

**Conclusion:** CHM improved the size and severity of the skin lesions and sleep quality in patients with AD. Comparing the adverse effects between the two groups, CHM is safe. However, CHM does not improve the quality of life or the patient’s IgE levels.

## 1 Introduction

Atopic dermatitis (AD) is a dermatological disease characterized by chronic inflammation (Damour, et al., 2020). Studies have found that more than 20% of children and 3% of adults worldwide suffer from atopic dermatitis (Nutten, 2015). Epidemiological studies in the United States and Europe over the past 20 years have shown a prevalence of atopic dermatitis of up to 25%, with an increasing trend over the years (Sybilski, et al., 2015). The basic clinical features are intensely pruritic dry, or eczema-like skin lesions (Lacouture and Sibaud 2018). Patients with AD often have other atopic diseases such as allergic rhinitis and asthma. These conditions can lead to sleep deprivation, depression, anxiety, social embarrassment, and other psychosocial concerns (Nutten, 2015).

The etiology of AD is unclear. Genetic factors, allergen stimulation (food, inhalation, etc.), autoantigens, infection, or skin dysfunction are all associated with the development of AD (Avena-Woods, 2017). In healthy humans, there is a dynamic balance in the Th1/Th2 cells. For AD patients with AD, there is an imbalance in the Th1/Th2 cells. This has emerged as an important mechanism in the pathogenesis of AD and is thought to be an important factor in disease progression. This imbalance is mainly caused by the overexpression of Th2 cytokines such as IL-4, IL-13, and IL-31 (Huang, et al., 2013). Secretion of thymic stromal lymphopoietin (TSLP) by epithelial cells, expressed in epithelial and stromal cells, is also a major allergenic cytokine in AD (Wilson, et al., 2013). Additionally, IgE is considered a hallmark antibody for allergic diseases such as AD. IgE is elevated in approximately 50%–80% of patients with atopic dermatitis and is one of the indicators for the diagnosis of atopic dermatitis (Furue, et al., 2017).

Traditional treatments for AD include antihistamines, topical corticosteroids, and immunosuppressants (Czarnowicki, et al., 2019). However, antihistamines are unsatisfactory for treating pruritus (Gholyaf, et al., 2020), and glucocorticoids cannot be used long-term because of their potential side effects (Ronchetti, et al., 2021), biologics are expensive, and relapse is common after withdrawal. Because of the chronic and relapsing nature of AD, treatment must be effective and have few side effects.

Chinese herbal medicine (CHM) has considerable benefits and low toxicity for the treatment of skin diseases and has a complete theoretical basis (Luo et al., 2021). Currently, there is growing interest in Chinese herbal medicine (CHM) as a potential complementary and alternative therapy (Cao, et al., 2015). The treatment of atopic dermatitis with CHM is diverse and mainly divided into internal and external treatments, all of which are reported to have good efficacy (Lin, et al., 2019). This may be related to the that many herbs and their active ingredients have anti-inflammatory, anti-allergic, antioxidant, and anti-angiogenic effects, and can mitigate the effects of AD by restoring the skin barrier while balancing Th1/Th2 cell levels and regulating the expression of cytokines and chemokines through a variety of mechanisms with few side effects (Yan, et al., 2020).

CHM has also been questioned as complementary and alternative medicine for the treatment of AD (Berke, et al., 2012). Several systematic reviews about the efficacy of CHM in the treatment of AD have been published (Gu, et al., 2013; Tan, et al., 2013). However, all of them lack high-level clinical evidence and a substantial amount of new trials have been published. So, this review aimed to systematically evaluate the efficacy and safety of CHM as a treatment for AD, based on high-quality clinical studies.

## 2 Methods

This study was conducted according to the Cochrane Handbook on Systematic Review of Interventions and Preferred Reporting Items for Systematic Review and Meta-Analysis (PRISMA) guidelines (Liberati, et al., 2009) (Supplementary Table S1).

### 2.1 Search strategy

We searched seven databases, PubMed, Embase, Cochrane Library, Web of Science, China National Knowledge Infrastructure (CNKI), the Chongqing VIP Chinese Science (VIP), and Wanfang Database from inception to 15 February 2022. We used free text words for the search in the title and abstract. The search formula is as follows (“Atopic dermatitis” OR “Eczema” OR “Dermatitis”) and (“Traditional Chinese Medicine” OR “Chinese drug” OR “Chinese herbal drug” OR “herbal”) and (“Clinical” OR “trial” OR “Clinical trial”) (Supplementary Table S2).

### 2.2 Inclusion and exclusion criteria

The inclusion criteria for the study were: 1) patients diagnosed according to diagnostic criteria; 2) randomized controlled trials (RCTs) comparing CHM with placebo for AD, 3) RCTs with a Jadad score ≥4 (Jadad, et al., 1996), and 4) studies must report adverse effects and efficacy outcome indicators.

The exclusion criteria were 1) the two groups had different modes of drug delivery method, and 2) combination intervention using drugs other than CHM in the trial group.

### 2.3 Data extraction

Two researchers (X.C. Cai, and X.Y. Sun) carefully screened eligible articles based on the inclusion and exclusion criteria. Two researchers (S.G. Hong and J. Wang) completed separate tables for data extraction, including author name, publication year, sample characteristics (size, age, sex, and duration), interventions in the experiment (specific drug names, composition, and dosage), and control groups. The inconsistencies were confirmed by a third investigator (L. Liu).

### 2.4 Outcome measures

The primary outcome was the number of patients whose EASI scores decreased by 50%/75%/90% or more from baseline. EASI scores is a composite score that allows the assessment of lesion severity and area. A dermatologist evaluated the clinical presentation score (erythema, induration or population, excoriation, and lichenification), lesion area size score, and body surface area score and then calculated the EASI score. Scoring Atopic Dermatitis (SCORAD) clinical tool, which includes the assessment of clinical signs and symptoms and the patient’s self-assessment of pruritus and sleep, was used as a secondary outcome. Other secondary outcomes included body surface area (BSA), surface damage score, Investigator’s Global Assessment decreased by 1/2 points (IGA 1/IGA 2), Dermatology Quality of Life Index/Children’s Dermatology Quality of Life Index (DLQI/CDLQI), visual analog scale (VAS), sleep score, IgE, and adverse events (AEs).

### 2.5 Risk of bias

To assess the quality of the clinical trials, two investigators (J.L.Chen, and M. Zhang) completed the Jadad scale for each of the included studies. When the two authors disagreed, a third investigator (C.X. Wang) was consulted. The Jadad scale has four dimensions (randomization, concealment, blind method, and reports of withdrawals), with a total of seven scores. Trials scoring ≥ 4 points were considered high quality (Jadad, et al., 1996).

### 2.6 Statistical analysis

We used RevMan5.3 software provided by the Cochrane Collaboration, to synthesize the results of the meta-analysis. When we extracted date from the included articles and found that several articles report three levels of EASI score (90/75/50), for which we set up a subgroup analysis to obtain more complete results. Risk ratios (RR) with 95% confidence intervals (CI) were assessed for dichotomous data, continuous data, mean differences (MD), and standard mean differences (SMD). Throughout the experiment, if homogeneity was present (*p* > 0.1, I^2^<50%), a fixed-effects model was used; otherwise, a random-effects model was used. Statistical significance was set at *p* < 0.05. Evidence level evaluation using GRADE Profiler 3.6.

## 3 Results

### 3.1 Included studies

**FIGURE 1:**
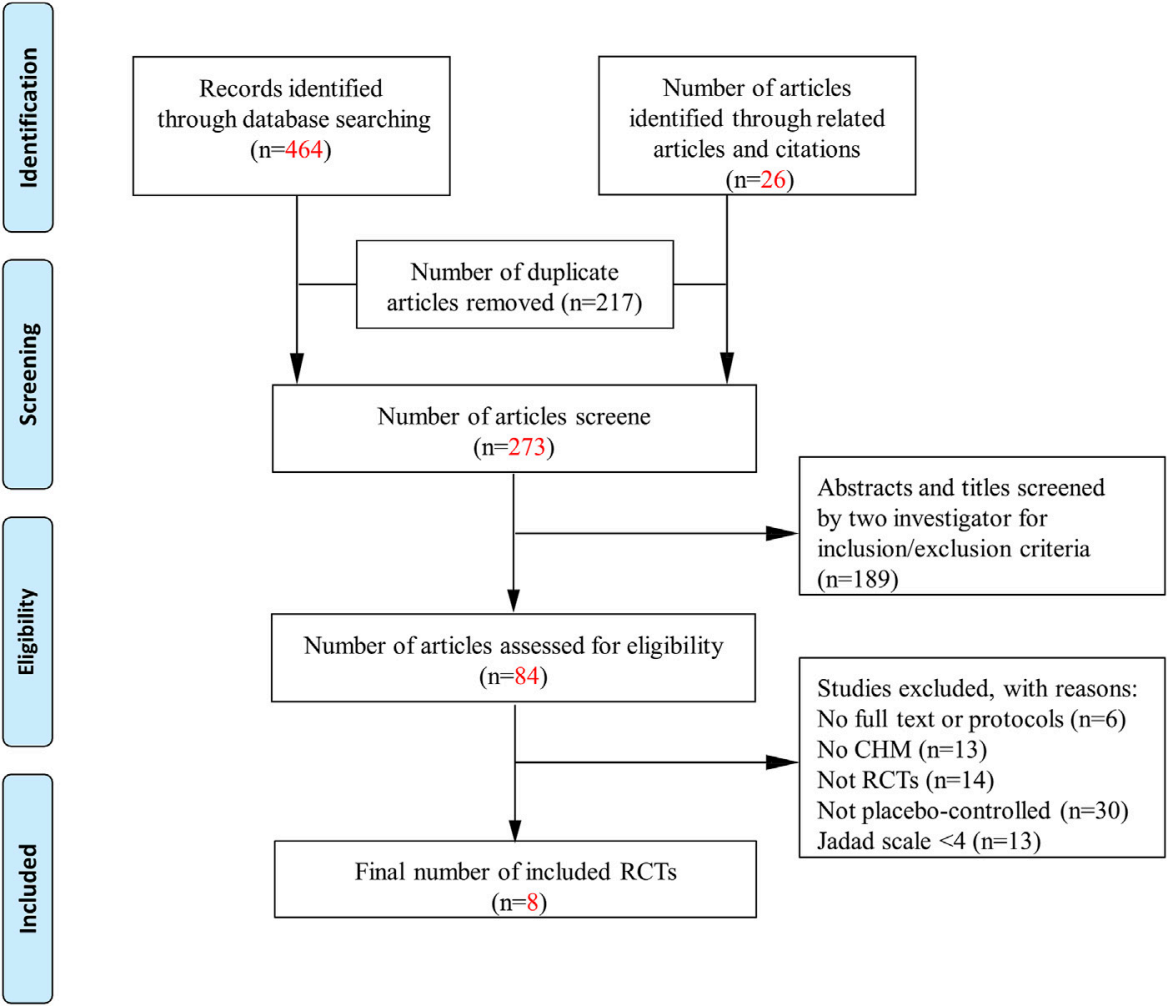
Flowchart. CHM, Chinese herbal medicine; RCTs: randomized control trials; AEs, adverse events.

After searching five databases, we initially identified 490 studies and 26 articles from relevant references. Of these, 217 duplicate articles were excluded and 189 irrelevant articles were removed after reviewing the title and abstract. Of the 84 remaining studies, 76 were excluded (six did not have full text or protocols, 13 used medicinal alternatives that were not CHM, 14 were not RCTs, and 30 were not placebo-controlled). Finally, the remaining 21 studies were assessed using the Jadad score, of which 8 studies met the inclusion criteria (Jadad score ≥4) (Hon, et al., 2007; Sun, 2009; Cheng, et al., 2011; Gu, et al., 2018; Huang, et al., 2019; Tian, 2019; Lin, et al., 2020; Liu, 2021). Five trials were published in English and three in Chinese. Figure 1 shows a flowchart of the screening process.

### 3.2 Study characteristics

In total, 662 patients (322 in the experimental group and 340 in the control group) were included. A placebo was used in the control group in all eight trials. However, the intervention drugs were not identical. Four trials (Sun, 2009; Cheng, et al., 2011; Gu, et al., 2018; Liu, 2021) used granules, two (Hon, et al., 2007; Huang, et al., 2019) used capsules, and one (Lin, et al., 2020) used ointments, and one (Tian, 2019) used liquids. The treatment duration varied from 4 to 12 weeks. Four (Sun, 2009; Tian, 2019; Lin, et al., 2020; Liu, 2021) reported the number of patients achieving EASI-90 at the end of treatment, three (Sun, 2009; Lin, et al., 2020; Liu, 2021) reported on EASI-75, and four (Sun, 2009; Tian, 2019; Lin, et al., 2020; Liu, 2021) reported on EASI-50, which signifies an EASI score reduction of at least 90, 75, and 50%, respectively. Three trials (Sun, 2009; Tian, 2019; Liu, 2021) assessed SCORAD, four (Sun, 2009; Tian, 2019; Lin, et al., 2020; Liu, 2021) measured the BSA score, and three (Sun, 2009; Tian, 2019; Liu, 2021) reported on the surface damage score, two (Huang, et al., 2019; Lin, et al., 2020) used IGA 1 or IGA 2, four trials (Gu, et al., 2018; Huang, et al., 2019; Tian, 2019; Lin, et al., 2020) assessed DLQI/CDLQI, five (Sun, 2009; Huang, et al., 2019; Tian, 2019; Lin, et al., 2020; Liu, 2021) measured VAS scores, two (Sun, 2009; Tian, 2019) reported sleep scores, and two (Cheng, et al., 2011; Liu, 2021) detected cytokine IgE levels. Adverse events were reported in all eight trials. The names of the CHMs, drug composition, doses, and dosages are listed in Table 1.

**TABLE 1:** Characteristics of the included trials.

Author year	Sample size		Age (years) (mean ± SD)		Gender (M/F)		Duration (mean ± SD)		Intervention		Course of treatment	Adverse events	
	E	C	E	C	E	C	E	C	E	C		E	C
Cheng et al. (2011)	46	23	12.2 ± 11.1	13.6 ± 6.4	25/21	12/11	8.4 ± 8.2	6.7 ± 6.8	CHM granules	Placebo	8 weeks	2	0
Huang et al. (2019)	120	118	50.0 ± 13.3	48.6 ± 13.7	58/62	51/67	64.0 ± 78.0	5.3 ± 6.5	CHM capsule	Placebo	4 weeks	21	22
Gu et al. (2018)	16	14	6–16	9/21	—	—	CHM granules	Placebo	12 weeks	1	2		
Hon et al. (2007)	42	43	11.7 ± 3.8	11.7 ± 3.5	23/19	23/20	—	—	CHM capsule	Placebo	12 weeks	29	19
Lin et al. (2019)	32	16	21.9 ± 7.4	23.0 ± 6.5	20/12	9/7	17.5 ± 6.4	18.2 ± 6.8	CHM ointment	Placebo	6 weeks	17	7
Sun, (2009)	14	11	9.86 ± 5.11	8.82 ± 3.55	6/8	6/5	—	—	CHM granules	Placebo	4 weeks	0	0
Liu (2021)	30	30	6.13 ± 1.94	6.5 ± 2.05	—	4.47 ± 1.97	4.30 ± 1.70	CHM granules	Placebo	4 weeks	0	0	
Tian (2019)	22	22	7.85 ± 2.08	8 ± 2.13	11/9	10/10	—	—	CHM liquid	Placebo	8 weeks	0	0

Author year	Name of herbs	Compositions	Usage
Cheng et al. (2011)	Xiao-Feng-San (XFS)	*Saposhnikovia divaricata (Turcz. ex Ledeb.) Schischk.* 2.5 mg*; Sesamum indicum L.* 2.5 mg*; Angelica sinensis (Oliv.) Diels* 2.5 mg; *Rehmannia glutinosa (Gaertn.) DC.* 2.5 mg*; Sophora flavescens Aiton* 2.5 mg*; Atractylodes lancea (Thunb.) DC.* 2.5 mg*; Tabernaemontana divaricata (L.) R.Br. ex Roem. & Schult.* 2.5 mg*; Sesamum indicum L.* 2.5 mg*; Anemarrhena asphodeloides Bunge* 2.5 mg*; OrthosipHon et al. aristatus* var. *aristatus* 2.5 mg*; Akebia trifoliata (Thunb.) Koidz.*1.25 mg*; Glycyrrhiza glabra L.*1.25 mg*; Arctium lappa L.* 2.5 mg	3–7 years 3 g; 8–12 years 6 g; >13 9 g; tid
Huang et al. (2019)	Run Zao Zhi Yang (RZZYC)	*Rehmannia glutinosa (Gaertn.) DC.; Sophora flavescens Aiton; Reynoutria multiflora (Thunb.) Moldenke; Urtica dentata Hand.-Mazz.; Morus alba L.”*	4 capsules tid
Gu et al. (2018)	Pei Tu Qing Xin (PTQX)	*Glycyrrhiza glabra L.; Angelica dahurica (Hoffm.) Benth. & Hook.f. ex Franch. & Sav.; Pseudostellaria heterophylla (Miq.) Pax; Dioscorea oppositifolia L.; Coix lacryma-jobi L.; Imperata cyLin et al. drica (L.) P.Beauv.; Forsythia suspensa (Thunb.) Vahl; Forsythia suspensa (Thunb.) Vahl; Margarita Powder*	bid
Hon et al. (2007)	—	*Atractylodes lancea (Thunb.) DC.* 2 g*; Lonicera japonica Thunb.* 2 g*; Mentha canadensis L.* 1 g*; Paeonia × suffruticosa Andrews* 2 g*; Phellodendron amurense Rupr.* 2 g	3 capsules bid
Lin et al. (2019)	Indigo Ointment	*Indigofera tinctoria L.* 200 μg/g	0.5 g/100 cm^2^ bid
Sun, (2009)	Jian Pi Shen Shi	*Dioscorea oppositifolia L.; Coix lacryma-jobi L.; Codonopsis pilosula (Franch.) Nannf.; Alisma plantago-aquatica subsp. orientale (Sam.) Sam.; Smilax glabra Roxb.; Atractylodes macrocephala Koidz.; Ziziphus jujuba Mill.; Citrus × aurantium L.; Lablab purpureus subsp. Purpureus; Platycodon grandiflorus (Jacq.) ADC.*	3–11 years 6 g; 12–20 12 g; tid
Liu (2021)	Shen Lin et al. g Bai Zhu	*Glycyrrhiza glabra L.* 6 g*; Dioscorea oppositifolia L.* 10 g*; Coix lacryma-jobi L.* 15 g*; Codonopsis pilosula (Franch.) Nannf.* 10 g*; Smilax glabra Roxb.* 10 g; *Atractylodes macrocephala Koidz.* 10 g; *Ziziphus jujuba Mill.* 6 g; *Citrus × aurantium L.* 10 g; *Lablab purpureus subsp. Purpureus* 10 g; *Platycodon grandiflorus (Jacq.) A.DC.* 9 g; *Melia azedarach L.* 9 g	1 bag; bid
Tian (2019)	Jian Pi Zhu Yun	*Forsythia suspensa (Thunb.) Vahl* 15 g*; Smilax glabra Roxb.* 15 g*; Nephrolepis cordifolia (L.) C.Presl* 15 g*; Periploca forrestii Schltr.* 30g*; CONCHA OSTREAE* 30 g*; Ephedra sinica Stapf* 6 g	1pc; bid

SD: standard deviation; E: experimental; C: control; M: male; F: female; w: week; y: year; tid: ter in die; bid: bis in die; pc: piece.

### 3.3 Introduction of Chinese herbs

Forty-two herbs were used in the eight studies. More than three times use were *Glycyrrhiza* glabra L.; *Dioscorea* oppositifolia L.; Coix lacrymal-Jobi L.; Smilax glabra Roxb.

### 3.4 Risk of bias

Methodological quality was evaluated using Jadad scores, and the eight included trials were high-quality articles with Jadad scores ≥4. Sensitivity analysis was done for Jadad scores ≥4 and <4 (Supplementary Figure S1). All articles were performed by random and assigned concealment, but two studies (Sun, 2009; Huang, et al., 2019) did not report how the random sequence was generated, and three articles (Sun, 2009; Huang, et al., 2019; Liu, 2021) did not provide details of the concealment process. Although all articles utilized blinding, one article (Tian, 2019) did not specify whether the study was double-blinded, and one article (Sun, 2009) did not provide details regarding the placebo (Supplementary Table S3).

### 3.5 Primary outcome

#### 3.5.1 EASI 90/75/50

We performed a subgroup analysis of the different EASI score decline rates. The EASI-90 response rate was higher in the CHM group than in the placebo group (EASI-90: RR, 3.72; 95% CI, 1.76 to 7.83; *p* = 0.01) (Table 2; Supplementary Figure S2). For the EASI-75 and EASI-50, the results were not statistically significant (EASI-75: RR, 1.72; 95% CI, 1.12 to 2.66; *p* = 0.01; EASI-50: RR, 3.47; 95% CI, 2.28 to 3.85; *p* = 0.01).

**TABLE 2:** The primary outcome of efficacy between the CHM and placebo groups.

Trials	CHM		Placebo		RR [95%CI]	*p*-value
	Events	Total	Events	Total		
EASI score						
EASI-90						
Huang et al. (2019)	16	120	3	118	5.24 [1.57, 17.53]	
Lin et al. (2019)	4	32	0	16	4.64 [0.26, 81.16]	
Liu (2021)	9	30	4	30	2.25 [0.78, 6.52]	
Sun, (2009)	0	14	0	11	Not estimable	
Tian (2019)	2	20	0	20	5.00 [0.26, 98.00]	
Meta-analysis (I^2^ = 0%)					3.72 [1.76, 7.83]	0.01*
EASI-75						
Lin et al. (2019)	8	32	2	16	2.00 [0.48, 8.35]	
Liu (2021)	23	30	14	30	1.64 [1.07, 2.53]	
Sun, (2009)	1	14	0	11	2.40 [0.11, 53.77]	
Meta-analysis (I^2^ = 0%)					1.72 [1.12, 2.66]	0.01*
EASI-50						
Huang et al. (2019)	67	120	17	118	3.88 [2.43, 6.19]	
Lin et al. (2019)	19	32	4	16	2.38 [0.97, 5.82]	
Sun, (2009)	11	14	1	11	8.64 [1.31, 57.13]	
Tian (2019)	12	20	4	20	2.33 [1.13, 4.83]	
Meta-analysis (I^2^ = 0%)					3.47 [2.43, 4.96]	0.01*
Meta-analysis (Fixed, I^2^ = 17%)					2.96 [2.28, 3.85]	0.01*

EASI, eczema area, and severity index; EASI-90/75/50, 90%/75%/50% improvement in the EASI, score from baseLin et al. e; CI, confidence interval; RR, risk ratio. *p< 0.05.

### 3.6 Secondary outcome

#### 3.6.1 Scoring of atopic dermatitis (SCORAD)

Patients receiving CHM had a lower SCORAD than those taking placebo (MD, −10.20; 95% CI, −13.25 to −7.15; *p* = 0.01) (Table 3; Supplementary Figure S3).

**TABLE 3:** The secondary outcome of efficacy between the CHM and placebo groups.

Trials	CHM		Placebo		MD [95%CI]	*p*-value
	Mean	SD	Mean	SD		
SCORAD						
Liu (2021)	17.3	9.52	25.33	7.82	−8.03 [−12.44, −3.62]	
Sun, (2009)	15.86	6.68	26.51	7.24	−10.65 [−16.24, −5.06]	
Tian (2019)	16.88	11.17	31.08	10.54	−14.20 [−20.62, −7.78]	
Meta-analysis (Fixed, I^2^ = 18%)					−10.20 [−13.25, −7.15]	0.01*
SCORAD Indicator						
BSA						
Lin et al. (2019)	8.9	11.6	11.5	11.2	−2.60 [−9.40, 4.20]	
Liu (2021)	7.57	4.29	10.63	4.25	−3.06 [−5.22, −0.90]	
Sun, (2009)	6.78	7.25	11.95	9.78	−5.17 [−12.09, 1.75]	
Tian (2019)	2.33	1.87	3.8	2.56	−1.47 [−2.79, −0.15]	
Meta-analysis (I^2^ = 0%)					−2.01 [−3.11, −0.91]	0.01*
Surface damage score						
Liu (2021)	3.77	2.19	5.33	1.9	−1.56 [−2.60, −0.52]	
Sun, (2009)	2.43	1.36	3.55	1.21	−1.12 [−2.13, −0.11]	
Tian (2019)	11.2	8	19.78	7.72	−8.58 [−13.23, −3.93]	
Meta-analysis (I^2^ = 79%)					−2.25 [−4.17, −0.34]	0.02*
VAS						
Huang et al. (2019)	2.8	2.1	3.8	2.3	−1.00 [−1.50, −0.50]	
Lin et al. (2019)	5.2	2.2	5.5	2.6	−0.30 [−1.78, 1.18]	
Liu (2021)	2.27	1.42	4.53	1.63	−2.26 [−3.03, −1.49]	
Sun, (2009)	4.13	1.64	7.81	1.6	−3.68 [−4.96, −2.40]	
Tian (2019)	2.4	1.57	4.65	1.57	−2.25 [−3.18, −1.32]	
Meta-analysis (I^2^ = 83%)					−1.90 [−2.86, −0.93]	0.01*
Sleep score						
Sun, (2009)	1.85	1.75	5.91	2.43	−4.06 [−5.76, −2.36]	
Tian (2019)	0.35	1.43	2.85	1.63	−2.50 [−3.41, −1.59]	
Meta-analysis (I^2^ = 60%)					−3.11 [−4.60, −1.62]	0.01*
Meta-analysis (Random, I^2^ = 72%)					−2.16 [−2.79, −1.52]	0.01*

SCORAD, scoring atopic dermatitis; BSA, body surface area; VAS: visual analog scale; CI: confidence interval; MD: mean Deviation; RR: risk ratio. *p< 0.05.

#### 3.6.2 Body surface area (BSA)

Compared to the placebo group, BSA was lower in patients receiving CHM (MD, −2.01; 95% CI, −3.11 to −0.91; *p* = 0.01) (Table 3; Supplementary Figure S3).

#### 3.6.3 Surface damage score

The surface damage score was lower in patients receiving CHM than patients in the placebo group (MD, −2.25; 95% CI, −4.17 to −0.34; *p* = 0.02) (Table 3; Supplementary Figure S3).

#### 3.6.4 Visual analog scale (VAS)

The visual analog scale (VAS) was used to assess the pruritus severity. The CHM group had lower VAS scores than the control group (MD, −1.90; 95% CI, −2.86 to −0.93; *p* = 0.01) (Table 3; Supplementary Figure S3).

#### 3.6.5 Sleep score

Two trials applied sleep scores to assess the sleep quality of patients with AD. Our meta-analysis indicated that the CHM group had lower scores than did the control group (MD, −3.11; 95% CI, −4.60 to −1.62; *p* = 0.01) (Table 3; Supplementary Figure S3).

#### 3.6.6 DLQI/CDLQI

Four trials applied the Dermatology Life Quality Index/Children’s Dermatology Life Quality Index to assess the quality of life of patients with AD. The results suggested no significant difference between CHM and placebo (MD, -0.47; 95% CI, −1.30 to 0.37; *p* = 0.27) (Table 4; Supplementary Figure S3).

**TABLE 4:** Other secondary outcome of efficacy between the CHM and placebo groups.

Trials	CHM		Placebo		MD [95%CI]	*p*-value
	Mean	SD	Mean	SD		
DLQI/CDLQI						
Gu et al. (2018)	8.5	6.56	9.86	5.2	−1.36 [−5.57, 2.85]	
Huang et al. (2019)	2.95	3.2	3.27	3.78	−0.32 [−1.21, 0.57]	
Lin et al. (2019)	7.2	5.8	7.8	5.8	−0.60 [−4.08, 2.88]	
Tian (2019)	6.2	5.31	10	11.17	−3.80 [−8.97, 1.37]	
Meta-analysis (Fixed, I^2^ = 0%)					−0.47 [−1.30, 0.37]	0.27
IgE						
Cheng et al. (2011)	2,090	1,125.3	1,735	917.5	355.00 [−141.33, 851.33]	
Liu (2021)	1,899.5	345.3	2,309.8	438.6	−410.30 [−610.05, −210.55]	
Meta-analysis (Random, I^2^ = 87%)					-62.76 [−809.58, 684.05]	0.87
Trials	CHM		Placebo		RR [95%CI]	*p*-value
	Events	Total	Events	Total		
IGA						
IGA 2						
Huang et al. (2019)	72	120	23	118	3.08 [2.07, 4.57]	
Lin et al. (2019)	14	32	4	16	1.75 [0.69, 4.46]	
Meta-analysis (I^2^ = 16%)					2.83 [1.97, 4.06]	0.01*
IGA 1						
Huang et al. (2019)	72	120	23	118	3.08 [2.07, 4.57]	
Meta-analysis (Not applicable)					3.08 [2.07, 4.57]	0.01*
Meta-analysis (Fixed,I^2^ = 0%)					2.94 [2.25, 3.84]	0.01*

CDQLI: Children’s Dermatology Life Quality Index; DLQI: Dermatology life quality index; IGA, Investigator’s Global Assessment; IGA, 1/IGA, 2: Investigator’s Global Assessment decreased by 1/2 point; CI: confidence interval; MD: mean Deviation; RR: risk ratio. *p < 0.05.

#### 3.6.7 IgE

The IgE level is important in patients with AD. The results suggested no significant difference between CHM and placebo (MD, −62.76; 95% CI, −809.58 to 684.05; *p* = 0.87) (Table 4; Supplementary Figure S3).

#### 3.6.8 Investigator’s global assessment (IGA)

There were more patients in the herbal group with IGA2 (Investigator’s Global Assessment decreased by two points) than in the placebo group (RR, 2.83; 95% CI, 1.97–4.06; *p* = 0.01). For IGA1 (the Investigator’s Global Assessment decreased by 1 point), the number of patients in the CHM group was higher than that in the placebo group (RR, 3.08; 95% CI, 2.07–4.57; *p* = 0.01), and overall (RR, 2.94; 95% CI, 2.25–3.84; *p* = 0.01) (Table 4; Supplementary Figure S3).

#### 3.6.9 Safety

**FIGURE 2:**
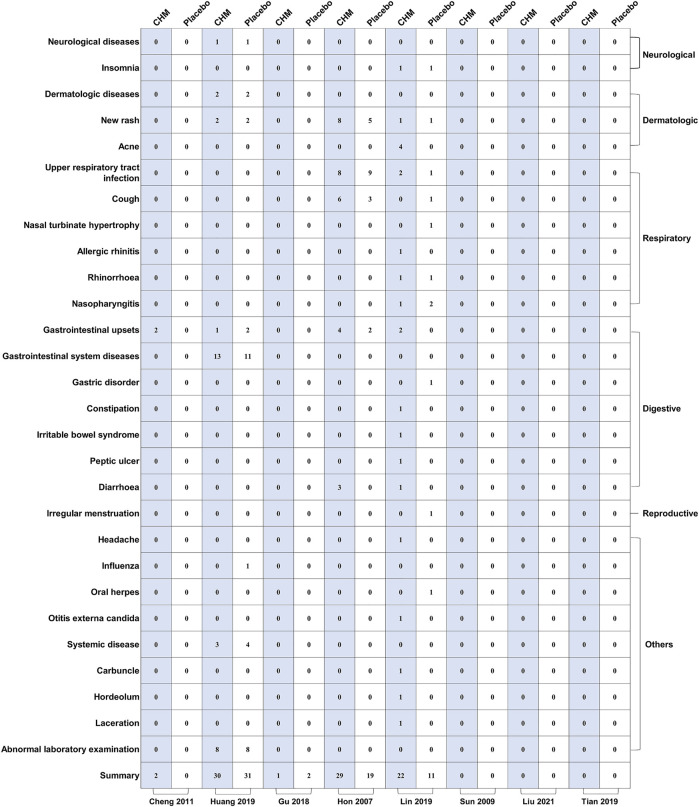
The mapping of specific adverse events between biological agents and control groups. MTX: methotrexate.

All trials assessed adverse events (AEs). The reported AEs included a new rash, upper respiratory tract infection, cough, gastrointestinal upsets, and diarrhea. Our results demonstrated that the incidence of AEs in patients treated with CHM was slightly higher than that in the placebo group (RR, 1.42; 95% CI, 1.06–1.90; *p* = 0.02) (Table 5; Supplementary Figure S4). We also classified the AEs into different body systems, including the neurological, dermatologic, respiratory, digestive, and reproductive systems (Figure 2).

**TABLE 5:** All reported adverse events.

Trials	CHM		Placebo		RR [95%CI]	*p*-value
	Events	Total	Events	Total		
AE						
Cheng et al. (2011)	2	46	0	23	2.55 [0.13, 51.09]	
Gu et al. (2018)	1	16	2	14	0.44 [0.04, 4.32]	
Hon et al. (2007)	29	42	19	43	1.56 [1.06, 2.31]	
Huang et al. (2019)	21	120	22	181	1.44 [0.83, 2.50]	
Lin et al. (2019)	17	32	7	16	1.21 [0.64, 2.31]	
Liu (2021)	0	30	0	30	Not estimable	
Sun, (2009)	0	14	0	11	Not estimable	
Tian (2019)	0	22	0	22	Not estimable	
Meta-analysis (Fixed, I^2^ = 0%)					1.42 [1.06, 1.90]	0.02*

AEs, adverse events; CI, confidence interval; RR, risk ratio; ^*^ *p* < 0.05.

We evaluated five adverse reactions: new rash (RR, 1.42; 95% CI, 0.61–3.33; *p* = 0.42), upper respiratory tract infection (RR, 0.92; 95% CI, 0.41–2.05; *p* = 0.84), cough (RR, 0.91; 95% CI, 0.09–8.89; *p* = 0.93), gastrointestinal upsets (RR, 1.77; 95% CI, 0.58–5.38; *p* = 0.32), and diarrhea (RR, 3.95; 95% CI, 0.52–30.12; *p* = 0.18) (Table 6; Supplementary Figure S5).

**TABLE 6:** Specific adverse events reported in the studies.

Trials	CHM		Placebo		MD [95%CI]	*p*-value
	Events	Total	Events	Total		
New rash						
Hon et al. (2007)	8	42	5	43	1.64 [0.58, 4.60]	
Huang et al. (2019)	2	120	2	181	1.51 [0.22, 10.56]	
Lin et al. (2019)	1	32	1	16	0.50 [0.03, 7.49]	
Meta-analysis (Fixed, I^2^ = 0%)					1.42 [0.61, 3.33]	0.42
Upper respiratory tract infection						
Hon et al. (2007)	8	42	9	43	0.91 [0.39, 2.13]	
Lin et al. (2019)	2	32	1	16	1.00 [0.10, 10.22]	
Meta-analysis (Fixed, I^2^ = 0%)					0.92 [0.41, 2.05]	0.84
Cough						
Hon et al. (2007)	6	42	3	43	2.05 [0.55, 7.66]	
Lin et al. (2019)	0	32	1	16	0.17 [0.01, 3.99]	
Meta-analysis (Random, I^2^ = 51%)					0.91 [0.09, 8.98]	0.93
Gastrointestinal upsets						
Cheng et al. (2011)	2	46	0	23	2.55 [0.13, 51.09]	
Hon et al. (2007)	4	42	2	43	2.05 [0.40, 10.59]	
Huang et al. (2019)	1	120	2	181	0.75 [0.07, 8.23]	
Lin et al. (2019)	2	32	0	16	2.58 [0.13, 50.68]	
Meta-analysis (Fixed, I^2^ = 0%)					1.77 [0.58, 5.38]	0.32
Diarrhoea						
Hon et al. (2007)	3	42	0	43	7.16 [0.38, 134.58]	
Lin et al. (2019)	1	32	0	16	1.55 [0.07, 35.94]	
Meta-analysis (Fixed, I^2^ = 0%)					3.95 [0.52, 30.12]	0.18

MD: mean Deviation; CI: confidence interval; RR: risk ratio; ^*^ *p* < 0.05.

#### 3.6.10 Evidence level

EASI-50, DLQI/CDLQI, VAS, New rush, Cough, gastrointestinal upsets, and total AE were moderate. Because the IgE result inconsistency was very serious, so the rating is low, and the rest are of high quality (Supplementary Figure S6).

## 4 Discussion

This systematic review included eight high-quality RCTs that evaluated the efficacy and safety of CHM for AD. Our study data showed an improvement in the CHM group compared to the placebo group. For the EASI-90, the arrival rate in the CHM group was higher (RR, 3.72) than that in the placebo group. This indicates that herbs are more likely to clear AD lesions than placebos are. In addition, other efficacy indicators such as SCORAD (RR, −10.20), BSA (RR, −2.01), surface damage score (RR, −2.25), VAS (RR, −1.90), sleep score (RR, −2.16), and IGA (RR, 2.94) improved to different degrees in the CHM group. These results indicate that Chinese medicine has better efficacy than placebo in many respects.

However, our systematic evaluation showed that CHM did not improve the DLQI/CDLQI (RR, −0.47) or IgE (RR, −62.76) levels in patients with AD. A possible reason for the lack of improvement in quality of life following CHM treatment may be that the patients reported only mild to moderate eczema that did not significantly impact their lives and work. Moreover, basic management strategies, such as the application of moisturizing creams, may reduce pruritus to some extent (Huang, et al., 2019). Cell-mediated immunity is also involved in AD pathogenesis. However, only two articles reported IgE levels and high heterogeneity (I^2^ = 87) in this analysis. These results indicate that herbal medicines did not reduce IgE.

Some studies have shown that certain herbs have anti-inflammatory effects. For example, the alcoholic extract of Coix lacrymal-Jobi L. has been shown to reduce the cellular secretion of inflammatory factors IL-4, IL-6, and TNF-α, and inhibit allergic reactions by regulating the expression of the ERK signaling pathway (Chen, et al., 2010). The volatile oil of agrimony inhibits the NF-κB pathway and reduces the inflammatory response. Platycodon grandiflorus (Jacq.) A. DC. contains platycodon saponins, flavonoids, phenolic acids, and other chemicals with anti-inflammatory, antitumor, and other desirable pharmacological effects (Ting, 2017; Chen, et al., 2020). The exact mechanism of action of polyherbal preparations is unclear. This may explain the stochastic efficacy of herbal medicines in the regulation of the expression of inflammatory factors, and provides a marker for future new directions in the treatment of atopic dermatitis.

There was slightly higher in patients treated with CHM than in those treated with placebo. But no difference in the safety between the CHM and placebo groups in a new rash, upper respiratory tract infection, cough, gastrointestinal upsets, and diarrhea. Therefore, CHM can be safely used to treat AD.

This study has several limitations. First, sufficiently high-quality trials are limited. Moreover, even if a study showed that CHM is more effective than placebo, it was difficult to standardize the included trials in terms of drug composition, dose, and treatment course, which may have affected the validity of our results. Although we list the herbs that were used more than 3 times, it remains unclear which CHM or herbal combination should be used in the clinical setting.

## 5 Conclusion

In summary, CHM improved the size and severity of the skin lesions and sleep quality in patients with AD. All included studies reported adverse events. Comparing the adverse effects between the CHM and placebo groups, CHM was safe. However, CHM does not improve the quality of life or patients’ IgE levels.

## Data Availability

The original contributions presented in the study are included in the article/Supplementary Material, further inquiries can be directed to the corresponding authors.

## Abbreviations

AD, Atopic Dermatitis; AEs, adverse events; BSA, body surface area; CDQLI, Children’s Dermatology Life Quality Index; CHM, Chinese herbal medicine; CNKI, China national knowledge infrastructure; CI, confidence intervals; DLQI, Dermatology Life Quality Index; EASI, Eczema Area and Severity Index; EASI 50/75/90, EASI score decreases more than 50%/75%/90% from baseline; HD, High dose; IGA, Investigator’s Global Assessment; IGA 1/IGA 2, Investigator’s Global Assessment decreased by 1/2 point; IL-4, interleukin-4; IL-13, interleukin-13; IL-31, interleukin-31; LD, Low dose; MD, mean difference; MTX, methotrexate; PRISMA, Preferred Reporting Items for Systematic Review and Meta-Analysis; RCTs, randomized control trials; RR, Risk ratios; SCORAD, Scoring Atopic Dermatitis; SD: Standard Deviation; SMD, standard mean difference; T_H_1, T helper type 1 cells; T_H_2, T helper type 2 cells; TSLP, Thymic Stromal Lymphopoietin; VAS, visual analog scale.
