# Supplementary material for: Efficacy and safety of Chinese herbal medicine for atopic dermatitis: Evidence from eight high-quality randomized placebo-controlled trials
Source: Front Pharmacol. 2022 Sep 27;13:927304. doi: 10.3389/fphar.2022.927304 (PMC9551201; doi:10.3389/fphar.2022.927304)
Supplement: Supplementary file 2 [file Image6.pdf]

TCM versus Placebo for Atopic Dermatitis

01-Aug-2022

1 Efficacy

1.12 EASI score

Study or Subgroup	Treatment Mean	Treatment SD	Treatment Total	Control Mean	Control SD	Control Total	Weight	Mean Difference IV, Fixed, 95% CI	Mean Difference IV, Fixed, 95% CI	Risk of bias A B C D
Ahn 2018	5.17	3.47	11	3.51	3.21	12	2.0%	1.66 [-1.08, 4.40]		
Gu 2018	6.71	6.32	16	6.63	6.79	16	0.7%	0.08 [-4.47, 4.63]		
Yen 2016	0.83	0.53	16	1.01	0.99	15	47.3%	-0.18 [-0.74, 0.38]		
Huang 2019	2.42	3.07	120	4.02	3.65	118	20.5%	-1.60 [-2.46, -0.74]		
Ma 2020	3.24	2.91	81	4.99	4.16	82	12.4%	-1.75 [-2.85, -0.65]		
Sun 2009	26.51	5.05	14	28.67	9.34	11	0.4%	-2.16 [-8.28, 3.96]		
Lai 2017	5.86	2.54	45	8.95	2.16	45	15.9%	-3.09 [-4.06, -2.12]		
Lin 2019	6.4	6.5	32	9.8	7.9	16	0.8%	-3.40 [-7.88, 1.08]		
**Total (95% CI)**			**335**			**315**	**100.0%**	**-1.12 [-1.51, -0.73]**		

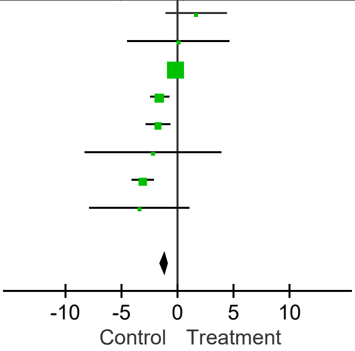

Heterogeneity: Chi² = 34.16, df = 7 (P < 0.0001); I² = 80%
Test for overall effect: Z = 5.66 (P < 0.00001)

Risk of bias legend
(**A**) Random sequence generation (selection bias)
(**B**) Allocation concealment (selection bias)
(**C**) Blinding of participants and personnel (performance bias)
(**D**) Blinding of outcome assessment (detection bias)
(**E**) Incomplete outcome data (attrition bias)
(**F**) Selective reporting (reporting bias)
(**G**) Other bias

TCM versus Placebo for Atopic Dermatitis 01-Aug-2022

1 Efficacy

1.12 EASI score

	Treatment			Control				Mean Difference	Risk of			
**Study or Subgroup**	**Mean**	**SD**	**Total**	**Mean**	**SD**	**Total**	**Weight**	**IV, Fixed, 95% CI**	**A**	**B**	**C**	**D**
Ahn 2018	5.17	3.47	11	3.51	3.21	12	0.0%	1.66 [-1.08, 4.40]				
Gu 2018	6.71	6.32	16	6.63	6.79	16	3.3%	0.08 [-4.47, 4.63]				
Yen 2016	0.83	0.53	16	1.01	0.99	15	0.0%	-0.18 [-0.74, 0.38]				
Huang 2019	2.42	3.07	120	4.02	3.65	118	91.6%	-1.60 [-2.46, -0.74]				
Ma 2020	3.24	2.91	81	4.99	4.16	82	0.0%	-1.75 [-2.85, -0.65]				
Sun 2009	26.51	5.05	14	28.67	9.34	11	1.8%	-2.16 [-8.28, 3.96]				
Lai 2017	5.86	2.54	45	8.95	2.16	45	0.0%	-3.09 [-4.06, -2.12]				
Lin 2019	6.4	6.5	32	9.8	7.9	16	3.4%	-3.40 [-7.88, 1.08]				
**Total (95% CI)**			**182**			**161**	**100.0%**	**-1.62 [-2.44, -0.80]**				

Heterogeneity: Chi² = 1.18, df = 3 (P = 0.76); I² = 0%

Test for overall effect: Z = 3.86 (P = 0.0001)

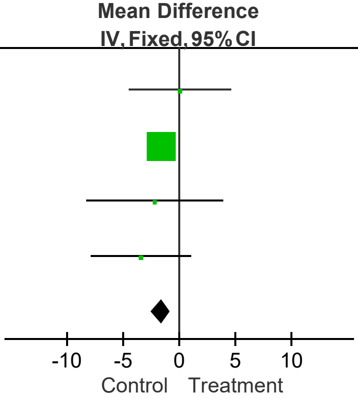

Risk of bias legend

(**A**) Random sequence generation (selection bias)

(**B**) Allocation concealment (selection bias)

(**C**) Blinding of participants and personnel (performance bias)

(**D**) Blinding of outcome assessment (detection bias)

(**E**) Incomplete outcome data (attrition bias)

(**F**) Selective reporting (reporting bias)

(**G**) Other bias
