# Supplementary figures and images for: Efficacy and safety of Chinese herbal medicine for atopic dermatitis: Evidence from eight high-quality randomized placebo-controlled trials
Source: Front Pharmacol. 2022 Sep 27;13:927304. doi: 10.3389/fphar.2022.927304 (PMC9551201; doi:10.3389/fphar.2022.927304)

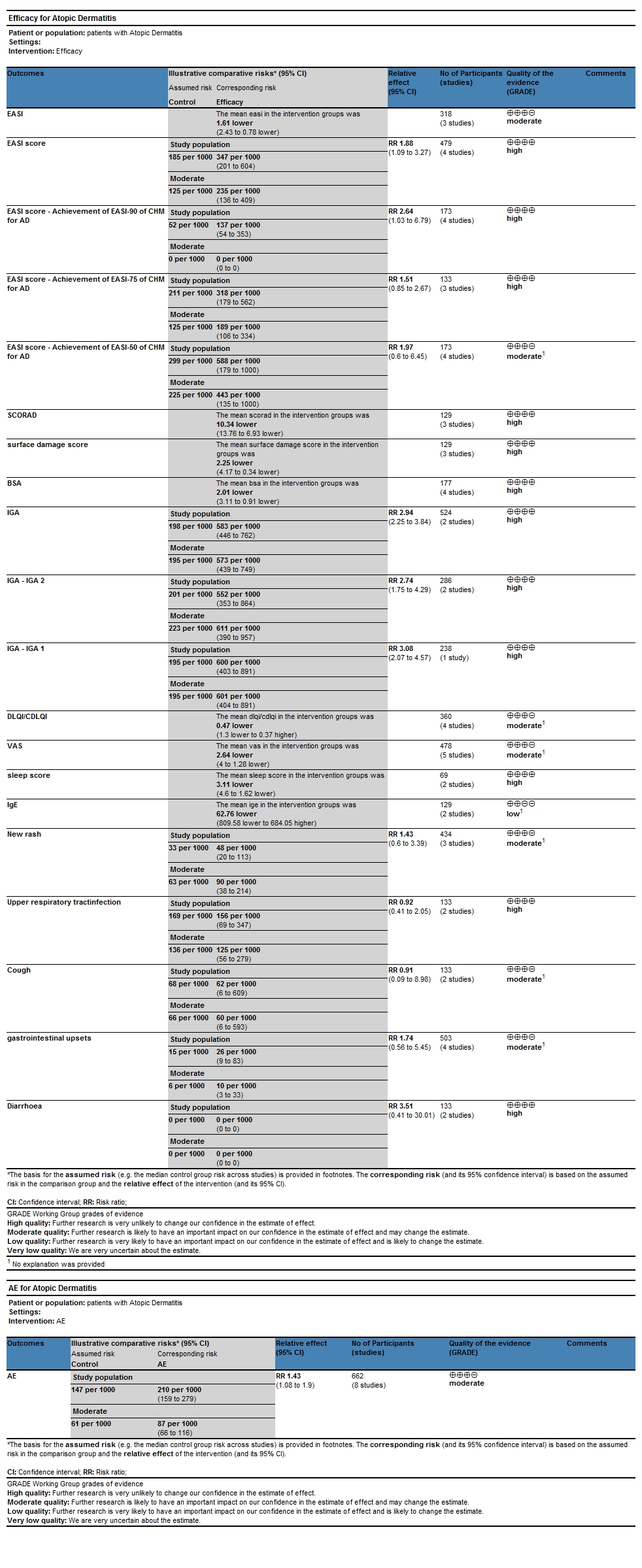

Supplement: Supplementary file 3 [file Image5.jpeg]
