# Supplementary material for: Efficacy and safety of Chinese herbal medicine for atopic dermatitis: Evidence from eight high-quality randomized placebo-controlled trials
Source: Front Pharmacol. 2022 Sep 27;13:927304. doi: 10.3389/fphar.2022.927304 (PMC9551201; doi:10.3389/fphar.2022.927304)
Supplement: Supplementary file 4 [file Image4.pdf]

1.7 New rash

	CHM		Placebo			Risk Ratio	Risk Ratio	Risk of Bias
**Study or Subgroup**	**Events**	**Total**	**Events**	**Total**	**Weight**	**M-H, Fixed, 95% CI**	**M-H, Fixed, 95% CI**	**A B C D E F G**
Hon 2007	8	42	5	43	62.8%	1.64 [0.58, 4.60]		
Huang 2019	2	120	2	181	20.3%	1.51 [0.22, 10.56]		
Lin 2019	1	32	1	16	16.9%	0.50 [0.03, 7.49]		
**Total (95% CI)**		**194**		**240**	**100.0%**	**1.42 [0.61, 3.33]**		
Total events	11		8					

Heterogeneity: Chi² = 0.65, df = 2 (P = 0.72); I² = 0%
Test for overall effect: Z = 0.81 (P = 0.42)

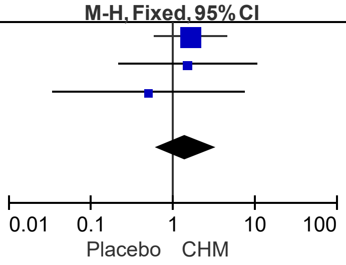

Risk of bias legend
(**A**) Random sequence generation (selection bias)
(**B**) Allocation concealment (selection bias)
(**C**) Blinding of participants and personnel (performance bias)
(**D**) Blinding of outcome assessment (detection bias)
(**E**) Incomplete outcome data (attrition bias)
(**F**) Selective reporting (reporting bias)
(**G**) Other bias

1.8 Upper respiratory tractinfection

	CHM		Placebo			Risk Ratio	Risk Ratio	Risk of Bias
**Study or Subgroup**	**Events**	**Total**	**Events**	**Total**	**Weight**	**M-H, Fixed, 95% CI**	**M-H, Fixed, 95% CI**	**A B C D E F G**
Hon 2007	8	42	9	43	87.0%	0.91 [0.39, 2.13]		
Lin 2019	2	32	1	16	13.0%	1.00 [0.10, 10.22]		
**Total (95% CI)**		**74**		**59**	**100.0%**	**0.92 [0.41, 2.05]**		
Total events	10		10					

Heterogeneity: Chi² = 0.01, df = 1 (P = 0.94); I² = 0%
Test for overall effect: Z = 0.20 (P = 0.84)

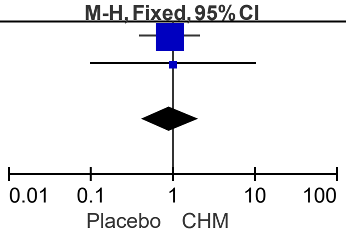

Risk of bias legend
(**A**) Random sequence generation (selection bias)
(**B**) Allocation concealment (selection bias)
(**C**) Blinding of participants and personnel (performance bias)
(**D**) Blinding of outcome assessment (detection bias)
(**E**) Incomplete outcome data (attrition bias)
(**F**) Selective reporting (reporting bias)
(**G**) Other bias

1.9 Cough

Study or Subgroup	CHM Events	CHM Total	Placebo Events	Placebo Total	Weight	Risk Ratio M-H, Random, 95% CI
Hon 2007	6	42	3	43	67.1%	2.05 [0.55, 7.66]
Lin 2019	0	32	1	16	32.9%	0.17 [0.01, 3.99]
**Total (95% CI)**		**74**		**59**	**100.0%**	**0.91 [0.09, 8.98]**
Total events	6		4			

Heterogeneity: Tau² = 1.59; Chi² = 2.05, df = 1 (P = 0.15); I² = 51%
Test for overall effect: Z = 0.08 (P = 0.93)

Risk Ratio M-H, Random, 95% CI

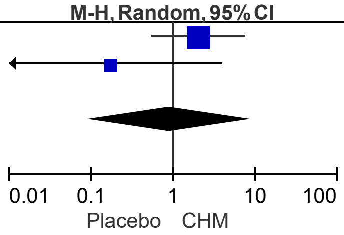

Risk of Bias
A B C D E F G

Risk of bias legend
(**A**) Random sequence generation (selection bias)
(**B**) Allocation concealment (selection bias)
(**C**) Blinding of participants and personnel (performance bias)
(**D**) Blinding of outcome assessment (detection bias)
(**E**) Incomplete outcome data (attrition bias)
(**F**) Selective reporting (reporting bias)
(**G**) Other bias

1.10 gastrointestinal upsets

Study or Subgroup	CHM Events	CHM Total	Placebo Events	Placebo Total	Weight	Risk Ratio M-H, Fixed, 95% CI
Cheng 2011	2	46	0	23	13.5%	2.55 [0.13, 51.09]
Hon 2007	4	42	2	43	40.4%	2.05 [0.40, 10.59]
Huang 2019	1	120	2	181	32.6%	0.75 [0.07, 8.23]
Lin 2019	2	32	0	16	13.5%	2.58 [0.13, 50.68]
**Total (95% CI)**		**240**		**263**	**100.0%**	**1.77 [0.58, 5.38]**
Total events	9		4			

Heterogeneity: Chi² = 0.64, df = 3 (P = 0.89); I² = 0%
Test for overall effect: Z = 1.00 (P = 0.32)

Risk Ratio M-H, Fixed, 95% CI

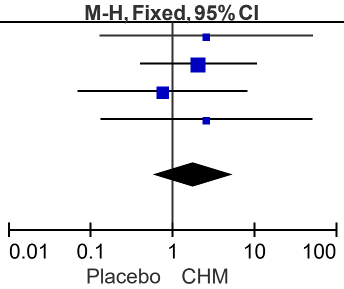

Risk of Bias
A B C D E F G

Risk of bias legend
(**A**) Random sequence generation (selection bias)
(**B**) Allocation concealment (selection bias)
(**C**) Blinding of participants and personnel (performance bias)
(**D**) Blinding of outcome assessment (detection bias)
(**E**) Incomplete outcome data (attrition bias)
(**F**) Selective reporting (reporting bias)
(**G**) Other bias

1.11 Diarrhoea

Study or Subgroup	CHM Events	CHM Total	Placebo Events	Placebo Total	Weight	Risk Ratio M-H, Fixed, 95% CI	Risk of Bias A B C D E F G
Hon 2007	3	42	0	43	42.8%	7.16 [0.38, 134.58]	
Lin 2019	1	32	0	16	57.2%	1.55 [0.07, 35.94]	
**Total (95% CI)**		**74**		**59**	**100.0%**	**3.95 [0.52, 30.12]**	
Total events	4		0				

Heterogeneity: Chi² = 0.50, df = 1 (P = 0.48); I^2 = 0%

Test for overall effect: Z = 1.33 (P = 0.18)

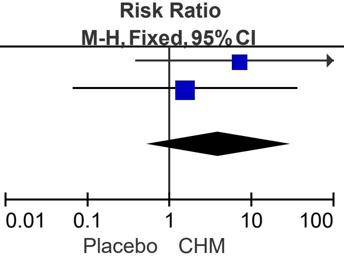

Risk of bias legend

(**A**) Random sequence generation (selection bias)
(**B**) Allocation concealment (selection bias)
(**C**) Blinding of participants and personnel (performance bias)
(**D**) Blinding of outcome assessment (detection bias)
(**E**) Incomplete outcome data (attrition bias)
(**F**) Selective reporting (reporting bias)
(**G**) Other bias
