# Supplementary material for: Efficacy and safety of Chinese herbal medicine for atopic dermatitis: Evidence from eight high-quality randomized placebo-controlled trials
Source: Front Pharmacol. 2022 Sep 27;13:927304. doi: 10.3389/fphar.2022.927304 (PMC9551201; doi:10.3389/fphar.2022.927304)
Supplement: Supplementary file 5 [file Image2.pdf]

1.2SCORAD

	CHM			Placebo				Mean Difference
**Study or Subgroup**	**Mean**	**SD**	**Total**	**Mean**	**SD**	**Total**	**Weight**	**IV, Fixed, 95% CI**
Liu 2021	17.3	9.52	30	25.33	7.82	30	47.7%	-8.03 [-12.44, -3.62]
Sun 2009	15.86	6.86	14	26.51	7.24	11	29.7%	-10.65 [-16.24, -5.06]
Tian 2019	16.88	11.17	22	31.08	10.54	22	22.5%	-14.20 [-20.62, -7.78]
**Total (95% CI)**			**66**			**63**	**100.0%**	**-10.20 [-13.25, -7.15]**

Heterogeneity: Chi² = 2.45, df = 2 (P = 0.29); I² = 18%
Test for overall effect: Z = 6.56 (P < 0.00001)

Mean Difference
IV, Fixed, 95% CI

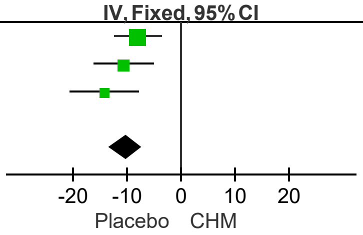

Ri
A B C

Risk of bias legend
(**A**) Random sequence generation (selection bias)
(**B**) Allocation concealment (selection bias)
(**C**) Blinding of participants and personnel (performance bias)
(**D**) Blinding of outcome assessment (detection bias)
(**E**) Incomplete outcome data (attrition bias)
(**F**) Selective reporting (reporting bias)
(**G**) Other bias

1.3 SCORAD Indicatior

Study or Subgroup	CHM Mean	CHM SD	CHM Total	Placebo Mean	Placebo SD	Placebo Total	Weight	Mean Difference IV, Random, 95% CI
**1.3.1 BSA**								
Lin 2019	8.9	11.6	32	11.5	11.2	16	0.8%	-2.60 [-9.40, 4.20]
Liu 2021	7.57	4.29	30	10.63	4.25	30	5.1%	-3.06 [-5.22, -0.90]
Sun 2009	6.78	7.25	14	11.95	9.78	11	0.8%	-5.17 [-12.09, 1.75]
Tian 2019	2.33	1.87	22	3.8	2.56	22	8.1%	-1.47 [-2.79, -0.15]
**Subtotal (95% CI)**			**98**			**79**	**14.9%**	**-2.01 [-3.11, -0.91]**
Heterogeneity: Tau² = 0.00; Chi² = 2.38, df = 3 (P = 0.50); I² = 0%								
Test for overall effect: Z = 3.57 (P = 0.0004)								
**1.3.2 surface damage score**								
Liu 2021	3.77	2.19	30	5.33	1.9	30	9.4%	-1.56 [-2.60, -0.52]
Sun 2009	2.43	1.36	14	3.55	1.21	11	9.5%	-1.12 [-2.13, -0.11]
Tian 2019	11.2	8	22	19.78	7.72	22	1.6%	-8.58 [-13.23, -3.93]
**Subtotal (95% CI)**			**66**			**63**	**20.6%**	**-2.25 [-4.17, -0.34]**
Heterogeneity: Tau² = 1.90; Chi² = 9.48, df = 2 (P = 0.009); I² = 79%								
Test for overall effect: Z = 2.31 (P = 0.02)								
**1.3.3 VAS**								
Huang 2019	2.8	2.1	120	3.8	2.3	181	11.6%	-1.00 [-1.50, -0.50]
Lin 2019	5.2	2.2	32	5.5	2.6	16	7.5%	-0.30 [-1.78, 1.18]
Liu 2021	2.27	1.42	30	4.53	1.63	30	10.6%	-2.26 [-3.03, -1.49]
Sun 2009	4.13	1.64	14	7.81	1.6	11	8.3%	-3.68 [-4.96, -2.40]
Tian 2019	2.4	1.57	22	4.65	1.57	22	9.9%	-2.25 [-3.18, -1.32]
**Subtotal (95% CI)**			**218**			**260**	**47.9%**	**-1.90 [-2.86, -0.93]**
Heterogeneity: Tau² = 0.95; Chi² = 23.25, df = 4 (P = 0.0001); I² = 83%								
Test for overall effect: Z = 3.84 (P = 0.0001)								
**1.3.4 sleep score**								
Sun 2009	1.85	1.75	14	5.91	2.43	11	6.6%	-4.06 [-5.76, -2.36]
Tian 2019	0.35	1.43	22	2.85	1.63	22	10.0%	-2.50 [-3.41, -1.59]
**Subtotal (95% CI)**			**36**			**33**	**16.6%**	**-3.11 [-4.60, -1.62]**
Heterogeneity: Tau² = 0.73; Chi² = 2.51, df = 1 (P = 0.11); I² = 60%								
Test for overall effect: Z = 4.09 (P < 0.0001)								
**Total (95% CI)**			**418**			**435**	**100.0%**	**-2.16 [-2.79, -1.52]**
Heterogeneity: Tau² = 0.84; Chi² = 45.89, df = 13 (P < 0.0001); I² = 72%								
Test for overall effect: Z = 6.65 (P < 0.00001)								
Test for subgroup differences: Chi² = 1.92, df = 3 (P = 0.59), I² = 0%								

Mean Difference IV, Random, 95% CI

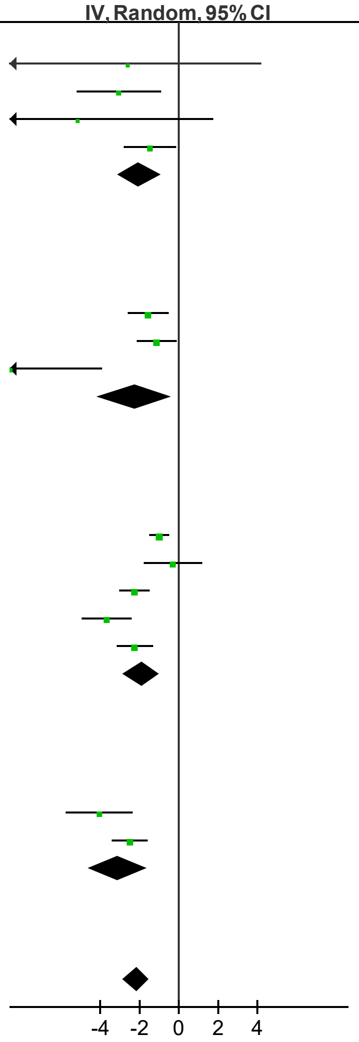

Risk of bias: A B C D

Risk of bias legend
(**A**) Random sequence generation (selection bias)
(**B**) Allocation concealment (selection bias)
(**C**) Blinding of participants and personnel (performance bias)
(**D**) Blinding of outcome assessment (detection bias)
(**E**) Incomplete outcome data (attrition bias)
(**F**) Selective reporting (reporting bias)
(**G**) Other bias

1.4DLQI/CDLQI

	CHM			Placebo				Mean Difference	Mean Difference	Risk of
**Study or Subgroup**	**Mean**	**SD**	**Total**	**Mean**	**SD**	**Total**	**Weight**	**IV, Fixed, 95% CI**	**IV, Fixed, 95% CI**	**A B C D**
Gu 2018	8.5	6.56	16	9.86	5.2	14	3.9%	-1.36 [-5.57, 2.85]		
Huang 2019	2.95	3.2	120	3.27	3.78	118	87.7%	-0.32 [-1.21, 0.57]		
Lin 2019	7.2	5.8	32	7.8	5.8	16	5.7%	-0.60 [-4.08, 2.88]		
Tian 2019	6.2	5.31	22	10	11.17	22	2.6%	-3.80 [-8.97, 1.37]		
**Total (95% CI)**			**190**			**170**	**100.0%**	**-0.47 [-1.30, 0.37]**		

Heterogeneity: Chi² = 1.88, df = 3 (P = 0.60); I² = 0%
Test for overall effect: Z = 1.10 (P = 0.27)

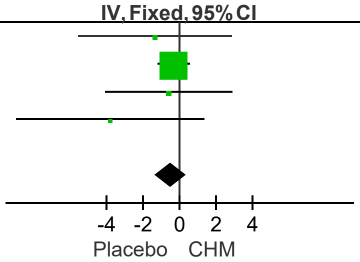

Risk of bias legend
(**A**) Random sequence generation (selection bias)
(**B**) Allocation concealment (selection bias)
(**C**) Blinding of participants and personnel (performance bias)
(**D**) Blinding of outcome assessment (detection bias)
(**E**) Incomplete outcome data (attrition bias)
(**F**) Selective reporting (reporting bias)
(**G**) Other bias

1.5 IGA

Study or Subgroup	CHM Events	CHM Total	Placebo Events	Placebo Total	Weight	Risk Ratio M-H, Fixed, 95% CI
**1.5.1 IGA 2**						
Huang 2019	72	120	23	118	44.8%	3.08 [2.07, 4.57]
Lin 2019	14	32	4	16	10.3%	1.75 [0.69, 4.46]
**Subtotal (95% CI)**		**152**		**134**	**55.2%**	**2.83 [1.97, 4.06]**
Total events	86		27			
Heterogeneity: Chi² = 1.19, df = 1 (P = 0.28); I² = 16%						
Test for overall effect: Z = 5.63 (P < 0.00001)						
**1.5.2 IGA 1**						
Huang 2019	72	120	23	118	44.8%	3.08 [2.07, 4.57]
**Subtotal (95% CI)**		**120**		**118**	**44.8%**	**3.08 [2.07, 4.57]**
Total events	72		23			
Heterogeneity: Not applicable						
Test for overall effect: Z = 5.58 (P < 0.00001)						
**Total (95% CI)**		**272**		**252**	**100.0%**	**2.94 [2.25, 3.84]**
Total events	158		50			
Heterogeneity: Chi² = 1.29, df = 2 (P = 0.53); I² = 0%						
Test for overall effect: Z = 7.93 (P < 0.00001)						
Test for subgroup differences: Chi² = 0.09, df = 1 (P = 0.76), I² = 0%						

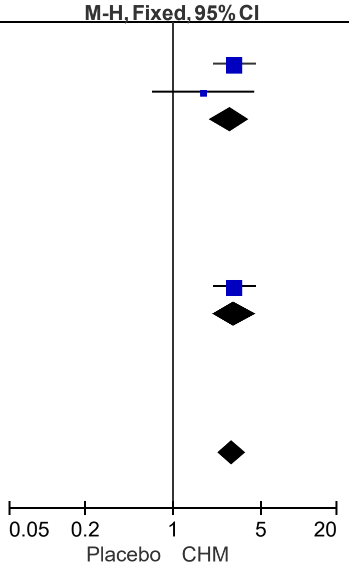

Risk of bias legend
(**A**) Random sequence generation (selection bias)
(**B**) Allocation concealment (selection bias)
(**C**) Blinding of participants and personnel (performance bias)
(**D**) Blinding of outcome assessment (detection bias)
(**E**) Incomplete outcome data (attrition bias)
(**F**) Selective reporting (reporting bias)
(**G**) Other bias

1.6 IgE

Study or Subgroup	CHM Mean	CHM SD	CHM Total	Placebo Mean	Placebo SD	Placebo Total	Weight	Mean Difference IV, Random, 95% CI
Cheng 2011	2,090	1,125.3	46	1,735	917.5	23	45.4%	355.00 [-141.33, 851.33]
Liu 2021	1,899.5	345.3	30	2,309.8	438.6	30	54.6%	-410.30 [-610.05, -210.55]
**Total (95% CI)**			**76**			**53**	**100.0%**	**-62.76 [-809.58, 684.05]**
Heterogeneity: Tau² = 255584.41; Chi² = 7.86, df = 1 (P = 0.005); I² = 87%								
Test for overall effect: Z = 0.16 (P = 0.87)								

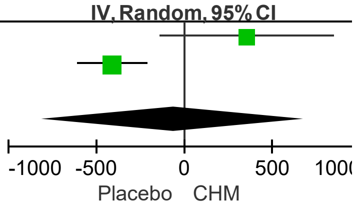

Risk of bias legend
(**A**) Random sequence generation (selection bias)
(**B**) Allocation concealment (selection bias)
(**C**) Blinding of participants and personnel (performance bias)
(**D**) Blinding of outcome assessment (detection bias)
(**E**) Incomplete outcome data (attrition bias)
(**F**) Selective reporting (reporting bias)
(**G**) Other bias
