# Supplementary material for: Efficacy and safety of Chinese herbal medicine for atopic dermatitis: Evidence from eight high-quality randomized placebo-controlled trials
Source: Front Pharmacol. 2022 Sep 27;13:927304. doi: 10.3389/fphar.2022.927304 (PMC9551201; doi:10.3389/fphar.2022.927304)
Supplement: Supplementary file 6 [file Image3.pdf]

2 AE

2.1 AE

Study or Subgroup	CHM Events	CHM Total	Placebo Events	Placebo Total	Weight	Risk Ratio M-H, Fixed, 95% CI	Risk of Bias A B C D E F G
Cheng 2011	2	46	0	23	1.4%	2.55 [0.13, 51.09]	
Gu 2018	1	16	2	14	4.4%	0.44 [0.04, 4.32]	
Hon 2007	29	42	19	43	38.8%	1.56 [1.06, 2.31]	
Huang 2019	21	120	22	181	36.2%	1.44 [0.83, 2.50]	
Lin 2019	17	32	7	16	19.3%	1.21 [0.64, 2.31]	
Liu 2021	0	30	0	30		Not estimable	
Sun 2009	0	14	0	11		Not estimable	
Tian 2019	0	22	0	22		Not estimable	
**Total (95% CI)**		**322**		**340**	**100.0%**	**1.42 [1.06, 1.90]**	
Total events	70		50				

Heterogeneity: Chi² = 1.62, df = 4 (P = 0.80); I² = 0%

Test for overall effect: Z = 2.33 (P = 0.02)

Risk Ratio M-H, Fixed, 95% CI

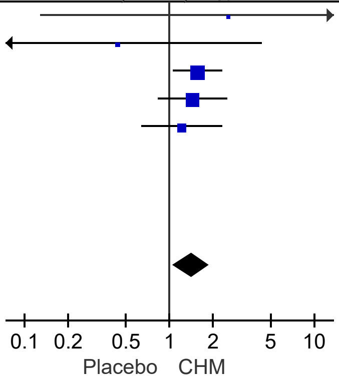

Risk of bias legend

(**A**) Random sequence generation (selection bias)

(**B**) Allocation concealment (selection bias)

(**C**) Blinding of participants and personnel (performance bias)

(**D**) Blinding of outcome assessment (detection bias)

(**E**) Incomplete outcome data (attrition bias)

(**F**) Selective reporting (reporting bias)

(**G**) Other bias
