# Supplementary material for: Efficacy and safety of Chinese herbal medicine for atopic dermatitis: Evidence from eight high-quality randomized placebo-controlled trials
Source: Front Pharmacol. 2022 Sep 27;13:927304. doi: 10.3389/fphar.2022.927304 (PMC9551201; doi:10.3389/fphar.2022.927304)
Supplement: Supplementary file 7 [file Table2.docx]

**Table S2 Search strategy**

**2.1 PubMed**

| Query | Search term |
| --- | --- |
| #1 | ((Atopic dermatitis[Title/Abstract]) OR (Eczema[Title/Abstract])) OR (Dermatitis[Title/Abstract]) |
| #2 | (((Traditional Chinese Medicine[Title/Abstract]) OR (Chinese drug[Title/Abstract])) OR (Chinese herbal drug[Title/Abstract])) OR (herbal[Title/Abstract]) |
| #3 | ((Clinical[Title/Abstract]) OR (trial[Title/Abstract])) OR (Clinical trial[Title/Abstract]) |
| #4 | ((#1) AND (#2)) AND (#3) |

**2.2** **Embase**

| Query | Search term |
| --- | --- |
| #1 | 'atopic dermatitis'/exp OR 'atopic dermatitis' OR (atopic AND ('dermatitis'/exp OR dermatitis)) OR eczema:ab,ti OR dermatitis:ab,ti |
| #2 | 'atopic dermatitis'/exp OR 'atopic dermatitis' OR (atopic AND ('dermatitis'/exp OR dermatitis)) OR eczema:ab,ti OR dermatitis:ab,ti |
| #3 | 'clinical'/exp OR clinical OR trial:ab,ti OR 'clinical trial':ab,ti |
| #4 | ((#1) AND (#2)) AND (#3) |

**2.3** **Cochrane Library**

| Query | Search term |
| --- | --- |
| #1 | ("atopic dermatitis"):ti,ab,kw OR (Eczema):ti,ab,kw OR (Dermatitis):ti,ab,kw |
| #2 | (Traditional Chinese Medicine):ti,ab,kw OR (Chinese drug):ti,ab,kw OR (Chinese herbal drug):ti,ab,kw OR (herbal):ti,ab,kw |
| #3 | (Clinical):ti,ab,kw OR (trial):ti,ab,kw OR (Clinical trial):ti,ab,kw |
| #4 | ((#1) AND (#2)) AND (#3) |

**2.4** **Web of Science**

| Query | Search term |
| --- | --- |
| #1 | ((TS=(Atopic dermatitis)) OR TS=(Eczema)) OR TS=(Dermatitis) |
| #2 | (((TS=(Traditional Chinese Medicine)) OR TS=(Chinese drug)) OR TS=(Chinese herbal drug)) OR TS=(herbal) |
| #3 | ((TI=(Clinical)) OR TI=(trial)) OR TI=(Clinical trial) |
| #4 | ((#1) AND (#2)) AND (#3) |

**2.5** **China National Knowledge Infrastructure (CNKI), VIP, and Wanfang**

| Query | Search term |
| --- | --- |
| #1 | (Atopic dermatitis[Subject]) |
| #2 | (Traditional Chinese Medicine[Subject]) |
| #3 | (Clinical trial[Subject]) |
| #4 | ((#1) AND (#2)) AND (#3) |
