# Supplementary material for: Efficacy and safety of Chinese herbal medicine for atopic dermatitis: Evidence from eight high-quality randomized placebo-controlled trials
Source: Front Pharmacol. 2022 Sep 27;13:927304. doi: 10.3389/fphar.2022.927304 (PMC9551201; doi:10.3389/fphar.2022.927304)
Supplement: Supplementary file 8 [file Table3.docx]

**Table S3. Jadad scores of the included trials.**

| **Author Year** | **Jadad Scale** | | | | |
| --- | --- | --- | --- | --- | --- |
|  | **a** | **b** | **c** | **d** | **T** |
| **Cheng 2011** | 2 | 2 | 2 | 1 | 7 |
| **Huang 2019** | 1 | 1 | 2 | 1 | 5 |
| **Gu 2018** | 2 | 2 | 2 | 1 | 7 |
| **Hon 2007** | 2 | 2 | 2 | 1 | 7 |
| **Lin 2020** | 2 | 2 | 2 | 1 | 7 |
| **Sun 2009** | 1 | 1 | 1 | 1 | 4 |
| **Liu 2021** | 2 | 1 | 2 | 1 | 6 |
| **Tian 2019** | 2 | 2 | 0 | 1 | 5 |

Four dimensions(a,b,c,d) of the Jadad scale. T, total.

a (2 points): randomized, 1 point; method appropriate, 1 point.

b (2 points): allocation concealment, 1 point; method appropriate, 1 point.

c (2 points): double-blind, 1 point; method appropriate, 1 point.

d (1 point): reported withdrawals and the reasons.
