# Supplementary material for: Efficacy and safety of Chinese herbal medicine for atopic dermatitis: Evidence from eight high-quality randomized placebo-controlled trials
Source: Front Pharmacol. 2022 Sep 27;13:927304. doi: 10.3389/fphar.2022.927304 (PMC9551201; doi:10.3389/fphar.2022.927304)
Supplement: Supplementary file 9 [file Image1.pdf]

1.1 EASI score

Study or Subgroup	CHM Events	CHM Total	Placebo Events	Placebo Total	Weight	Risk Ratio M-H, Fixed, 95% CI
**1.1.1 Achievement of EASI-90 of CHM for AD**						
Huang 2019	16	120	3	118	5.5%	5.24 [1.57, 17.53]
Lin 2019	4	32	0	16	1.2%	4.64 [0.26, 81.16]
Liu 2021	9	30	4	30	7.3%	2.25 [0.78, 6.52]
Sun 2009	0	14	0	11		Not estimable
Tian 2019	2	20	0	20	0.9%	5.00 [0.26, 98.00]
**Subtotal (95% CI)**		**216**		**195**	**14.9%**	**3.72 [1.76, 7.83]**
Total events	31		7			
Heterogeneity: Chi² = 1.23, df = 3 (P = 0.75); I² = 0%						
Test for overall effect: Z = 3.45 (P = 0.0006)						
**1.1.2 Achievement of EASI-75 of CHM for AD**						
Lin 2019	8	32	2	16	4.8%	2.00 [0.48, 8.35]
Liu 2021	23	30	14	30	25.5%	1.64 [1.07, 2.53]
Sun 2009	1	14	0	11	1.0%	2.40 [0.11, 53.77]
**Subtotal (95% CI)**		**76**		**57**	**31.3%**	**1.72 [1.12, 2.66]**
Total events	32		16			
Heterogeneity: Chi² = 0.13, df = 2 (P = 0.94); I² = 0%						
Test for overall effect: Z = 2.46 (P = 0.01)						
**1.1.3 Achievement of EASI-50 of CHM for AD**						
Huang 2019	67	120	17	118	31.2%	3.88 [2.43, 6.19]
Lin 2019	19	32	4	16	9.7%	2.38 [0.97, 5.82]
Sun 2009	11	14	1	11	2.0%	8.64 [1.31, 57.13]
Tian 2019	14	20	6	20	10.9%	2.33 [1.13, 4.83]
**Subtotal (95% CI)**		**186**		**165**	**53.8%**	**3.47 [2.43, 4.96]**
Total events	111		28			
Heterogeneity: Chi² = 2.94, df = 3 (P = 0.40); I² = 0%						
Test for overall effect: Z = 6.85 (P < 0.00001)						
**Total (95% CI)**		**478**		**417**	**100.0%**	**2.96 [2.28, 3.85]**
Total events	174		51			
Heterogeneity: Chi² = 11.99, df = 10 (P = 0.29); I² = 17%						
Test for overall effect: Z = 8.08 (P < 0.00001)						
Test for subgroup differences: Chi² = 6.75, df = 2 (P = 0.03), I² = 70.4%						

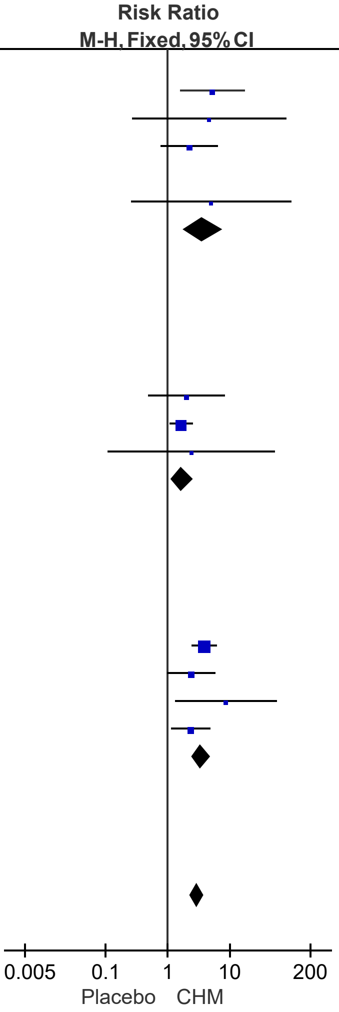

Risk of Bias
A B C D E F G

Risk of bias legend
(**A**) Random sequence generation (selection bias)
(**B**) Allocation concealment (selection bias)
(**C**) Blinding of participants and personnel (performance bias)
(**D**) Blinding of outcome assessment (detection bias)
(**E**) Incomplete outcome data (attrition bias)
(**F**) Selective reporting (reporting bias)
(**G**) Other bias
